# Editorial: 15 years of Frontiers in Cellular Neuroscience: the role of glial cells in schizophrenia and other related disorders

**DOI:** 10.3389/fncel.2024.1471266

**Published:** 2024-09-24

**Authors:** Hai-Ying Shen

**Affiliations:** Department of Pediatrics Neurology, College of Medicine, University of Nebraska Medical Center, Omaha, NE, United States

**Keywords:** glial cell, microglial, astrocyte, schizophrenia, neuroinflammation, neuropsychiatric disorders

## Introduction

As we celebrate the 15th anniversary of Frontiers in Cellular Neuroscience, it is an opportune moment to reflect on the pivotal research contributions that have shaped our understanding of glial cells in schizophrenia and related disorders. Over the past decade and a half, this journal has been at the forefront of unveiling the complex roles that glial cells, particularly microglia and astrocytes, play in the pathophysiology of mental illnesses.

## The intricate dance of microglia and neurons in schizophrenia

Hartmann et al. delve into the interactions between microglia and neurons in schizophrenia. Their comprehensive review highlights how microglia, the brain's resident immune cells, engage in a delicate balance of neuroprotection and neuroinflammation, which can be disrupted in schizophrenia. The study underscores the dual role of microglia in either exacerbating or mitigating neuronal damage, depending on their states (Paolicelli et al., 2022). The authors argue that therapeutic strategies targeting specific microglial reaction states could offer new avenues for treating schizophrenia.

## Astrocytes: the unsung heroes of brain health

Astrocytes, another type of glial cell, have also been spotlighted for their crucial role in maintaining brain homeostasis and their involvement in mental disorders. While review articles provide a detailed account of how astrocytes contribute to the pathophysiology of schizophrenia (Laricchiuta et al., 2024), and discuss the astrocytic regulation of neurotransmitter systems, blood-brain barrier integrity, and synaptic function, all of which are often disrupted in schizophrenia (Verkhratsky et al., 2023; Notter, 2021), Yan et al. have further elucidated the heterogeneity of astrocytes and their distinct roles in various central nervous system (CNS) regions and pathological states, emphasizing the importance of these cells in both health and disease contexts.

## The impact of environmental stressors: high altitude and neuroinflammation

In addition to genetic and molecular factors, environmental stressors such as high altitude have been shown to exacerbate microglial and astrocyte reactions, a common feature in many neurological disorders, including schizophrenia. Liu et al.'s study on the effects of high altitude on traumatic brain injury (TBI) reveals that hypoxic conditions can significantly worsen neuroinflammatory responses. The research demonstrates that L-serine, an endogenous amino acid, can mitigate these effects by modulating microglial activation through the NFAT1 pathway. This finding not only sheds light on the potential for L-serine as a neuroprotective agent but also underscores the broader implications of environmental factors on brain health.

## Innovative approaches to schizophrenia treatment

Singer and Yee explored the inhibition of astrocytic glycine transporter-1 (GlyT1) as a potential therapeutic approach for ameliorating NMDA receptor hypofunction in schizophrenia. The authors discuss the dual nature of GlyT1 inhibition, considering both its potential benefits and drawbacks. Their research suggests that while inhibiting GlyT1 may enhance N-methyl-D-aspartate (NMDA) receptor function and improve cognitive deficits in schizophrenia, careful consideration of the associated risks is essential. This study highlights the complexity of targeting astrocytic functions for therapeutic purposes and underscores the need for nuanced approaches in developing effective treatments.

The insights gained from these studies highlight the multifaceted roles of glial cells in brain health and disease. As we look forward to the next decade of Frontiers in Cellular Neuroscience, several promising research directions emerge. (i) Microglial Modulation: Developing therapies that can precisely modulate microglial activation states to promote neuroprotection while minimizing neuroinflammation. (ii) Targeting Astrocyte Functions: Exploring astrocyte-targeted therapies to restore neurotransmitter balance and synaptic function in schizophrenia. (iii) Environmental Considerations: Investigating the impact of environmental stressors on glial cell function and developing strategies to mitigate their adverse effects. (iv) GlyT1 Inhibition: Further examining the therapeutic potential and risks of inhibiting astrocytic glycine transporter-1 to enhance NMDA receptor function in schizophrenia.

In conclusion, the past 15 years have seen remarkable advancements in our understanding of glial cells and their roles in schizophrenia and related disorders. The continued exploration of these cellular players holds great promise for developing more effective treatments and improving the lives of those affected by these debilitating conditions.
